# Proteomic analysis of insulin secretory granules in INS-1 cells by protein correlation profiling

**DOI:** 10.1007/s41048-018-0061-3

**Published:** 2018-08-29

**Authors:** Min Li, Wen Du, Maoge Zhou, Li Zheng, Eli Song, Junjie Hou

**Affiliations:** 1 National Laboratory of Biomacromolecules, CAS Center for Excellence in Biomacromolecules, Institute of Biophysics, Chinese Academy of Sciences, Beijing, 100101 China; 2 College of Life Sciences, University of Chinese Academy of Sciences, Beijing, 100049 China

**Keywords:** Insulin secretory granules (ISGs), Proteome, Label-free proteomics, Protein correlation profiling

## Abstract

**Abstract:**

Insulin secretory granules (ISGs), a group of distinguishing organelles in pancreatic β cells, are responsible for the storage and secretion of insulin to maintain blood glucose homeostasis. The molecular mechanisms of ISG biogenesis, maturation, transportation, and exocytosis are still largely unknown because the proteins involved in these distinct steps have not been fully identified. Subcellular fractionation by density gradient centrifugation has been successfully employed to analyze the proteomes of numerous organelles. However, use of this method to elucidate the ISG proteome is limited by co-fractionated contaminants because ISGs are very dynamic and have abundant exchanges or contacts with other organelles, such as the Golgi apparatus, lysosomes, and endosomes. In this study, we developed a new strategy for identifying ISG proteins by protein correlation profiling (PCP)-based proteomics, which included ISG purification by OptiPrep density gradient centrifugation, label-free quantitative proteome, and identification of ISG proteins by correlating fractionation profiles between candidates and known ISG markers. Using this approach, we were able to identify 81 ISG proteins. Among them, TM9SF3, a nine-transmembrane protein, was considered a high confidence ISG candidate protein highlighted in the PCP network. Further biochemical and immunofluorescence assays indicated that TM9SF3 localized in ISGs, suggesting that it is a potential new ISG marker.

**Graphical abstract:**

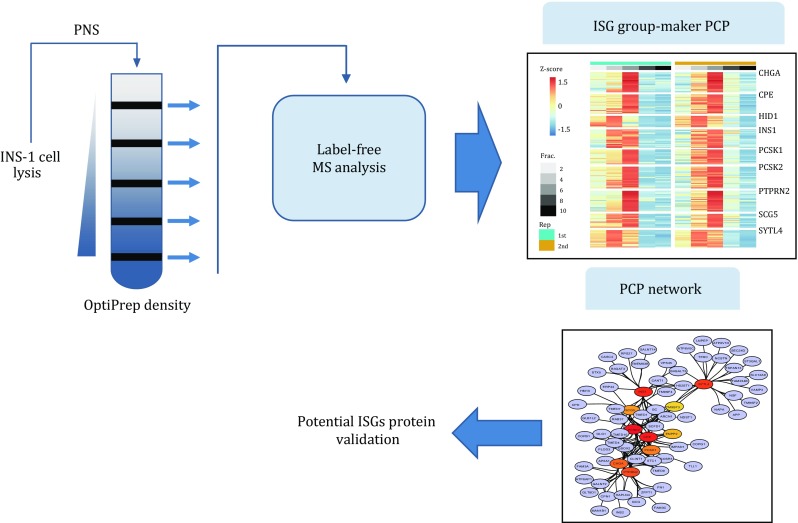

**Electronic supplementary material:**

The online version of this article (10.1007/s41048-018-0061-3) contains supplementary material, which is available to authorized users.

## Introduction

Insulin plays a crucial role in regulating energy balance and blood glucose homeostasis. Most insulin is synthesized, stored, and secreted by pancreatic β cells (Straub *et al.* 2004). In β cells, insulin and other secretory peptides are stored in a specialized organelle, namely, insulin secretory granules (ISGs), which serve as a storage pool. When the blood glucose level increases, ISGs fuse with the plasma membrane and release insulin. ISG biogenesis starts with budding at the trans-Golgi network (TGN), where proinsulin and other prohormones are sorted into immature insulin secretory granules (iISGs) (Arvan and Castle 1998; Arvan and Halban 2004). Then, iISGs undergo a precisely controlled maturation process to become mature ISGs (mISGs). The maturation process consists of several distinct steps, including homotypic fusion of iISGs, acidification of the granular lumen, proteolytic processing of prohormones into mature hormones, condensation of cargoes and removal of unwanted membrane and proteins. The resulting mISGs will be subsequently stored in a ready release pool or transported to the vicinity of the cell membrane, exhibiting specific responses to stimuli for regulated exocytosis (Du *et al.* 2016; Schvartz *et al.* 2012). However, due to lack of the sufficient understanding about the proteins involved in ISG-associated processes, including biogenesis, maturation, transportation, and exocytosis, the molecular mechanisms of these events are still largely unknown (Katsumata-Kato *et al.* 2015).

The map of organelle proteome is commonly achieved by subcellular fractionation methods, such as density gradient centrifugation and fluorescent-assisted organelle sorting (Lee *et al.* 2010). However, for ISGs, accurate proteome identification is a major challenge because ISGs are highly dynamic and have abundant exchanges or contacts with other organelles. Several studies have attempted to characterize the ISG proteome through proteomic analysis of ISG-enriched, biochemically isolated subcellular fractions. Brunner *et al*. (2007) identified 130 ISG proteins by using a two-step gradient purification of ISGs from INS-1E cells. In another study, Schvartz *et al*. (2012) used a three-step gradient purification procedure combined with stable isotope labeling with amino acids in cell culture (SILAC) to identify 140 proteins in mISGs. In addition, Hickey *et al*. (2009) employed density gradient centrifugation and fractionation and a further immuno-affinity approach, using antibodies specific to Vamp2 (vesicle-associated membrane protein 2), to enrich ISGs and finally identified 51 proteins in INS-1E cells. However, it was difficult to distinguish ISGs based on the size and the density of the granules by these analytical separation methods in previous studies (Tooze *et al.* 1991); thus, the interpretation of the proteomic data has been complicated by the presence of contaminant proteins from co-isolated organelles and/or membrane fragments, such as the Golgi apparatus, lysosomes and endosomes.

Protein correlation profiling (PCP)-based proteomics is an advantageous analytical method to identify multiprotein complexes or subcellular organelles, which are enriched by fractionation but not purified to homogeneity (Andersen *et al.* 2003; Kristensen *et al.* 2012). The basic principle of PCP analysis is to calculate the degree of similarity between the abundance profiles of candidate proteins with a “consensus profile”, defined as the average of the profiles of known marker proteins. A candidate with a high degree of similarity tends to be a protein of interest with strong potential. Recently, PCP studies have been successfully applied to investigate some subcellular organelles (Andersen *et al.* 2002, 2005; Foster *et al.* 2006; Krahmer *et al.* 2013), such as nucleolar and lipid droplets, but this approach has not previously been applied to ISGs.

We previously established an efficient two-step subcellular fractionation method for the enrichment of insulin granules from INS-1 cells, which involved OptiPrep gradient purification followed by Percoll solution purification. Using this method, the iISGs and mISGs can be successfully distinguished and enriched (Chen *et al.* 2015). In this study, we first separated the post-nuclear supernatant into five subcellular fractions by OptiPrep gradient centrifugation, and each fraction was then separated by SDS-PAGE, followed by in-gel digestion and mass spectrometry (MS) analysis. Purification profiles from ISG marker proteins quantified by MS intensity were used to define the consensus curves. Euclidean distance (ED) between the consensus curves and PCPs of all identified proteins was calculated and sorted. Finally, we considered 81 proteins in the top 100 ranked list that were identified repeatedly in both biological experiments as the ISG proteins. By constructing a PCP network analysis, TM9SF3, a 9-transmembrane protein, was highlighted as a high confidence ISG candidate protein. Further biochemical and immunofluorescence assays demonstrated that TM9SF3 co-localized with insulin, suggesting that it has the potential to be a new ISG marker.

## Experimental

### Materials and reagents

OptiPrep™ Density Gradient Medium, MgSO_4_, EDTA, HEPES, MES, and KOH were purchased from Sigma (St. Louis, MO, USA). EGTA was purchased from Amresco (USA). Sucrose was purchased from Beijing Biodee Biotechnology (Beijing, China).

The following antibodies were used for immunofluorescence and Western blot analysis: mouse monoclonal anti-proinsulin was purchased from HyTest (Turku, Finland). Guinea pig polyclonal antibody against insulin and rabbit monoclonal anti-TOMM20 antibody were purchased from Abcam (Cambridge, MA). Rabbit polyclonal anti-Vamp4, rabbit polyclonal anti-ATP6V1H, rabbit polyclonal anti-HID-1, rabbit polyclonal anti-CANX, rabbit polyclonal anti-CPE, rabbit polyclonal anti-SYTL4, and rabbit polyclonal anti-STX6 antibodies were purchased from Proteintech (Wuhan, China). Rabbit polyclonal anti-TM9SF3 antibody was purchased from Invitrogen (Carlsbad, CA, USA). Rabbit polyclonal anti-TM9SF3 antibody was purchased from Sigma (St. Louis, MO, USA).

### Cell culture

The INS-1 cell line was obtained from the Cell Resource Center, Peking Union Medical College (the headquarters of the National Infrastructure of Cell Line Resource, NSTI, Beijing, China) and was cultured in 1640 medium supplemented with 10% fetal bovine serum (Gibco 10270, South America), 1 mmol penicillin–streptomycin, and 100 μmol mercaptoethanol at 37 °C with 5% CO_2_.

### Optiprep separation of post-nuclear supernatant (PNS) from INS-1 cells

Separation of PNS was performed as described in a previous study (Chen *et al.* 2015) with minor modifications. In brief, INS-1 cells were grown on 15-cm-diameter dasher plate and washed twice with ice-cold PBS. Cells were harvested by trypsin and centrifuged at 1500 *g* for 10 min at 4 °C. The cell pellet was suspension with 2 ml homogenization buffer (0.3 mol/L sucrose, 1 mmol/L EDTA, 1 mmol/L MgSO_4_, 10 mmol/L MES-KOH, pH 6.5) containing 1:100 protease inhibitor (PI) by nitrogen cavitation for 20 min at 500 psi on ice. The optimized centrifugal speed was from 1000 to 5000 *g*. The cell lysis procedure not only removed unbroken cells and nuclear debris but also cell organelles and large complexes from the cell lysate by centrifuging at 5000 *g* for 15 min. The cell supernatant was collected and loaded on top of a discontinuous Optiprep gradient composed of five layers. The OptiPrep density gradient included five concentrations from top to bottom (8.8%, 13.2%, 17.6%, 23.4%, 30%), and the volume of each layer was 2 ml. Then, 30% OptiPrep was diluted with Buffer B (2 mmol/L EGTA, 20 mmol/L MES-KOH, pH 6.5), and the remaining solution was obtained by diluting 30% OptiPrep with Buffer A containing 1:100 PI in a SW40 tube. The sample was centrifuged at 100,000 *g* for 135 min, and the enrichment fraction was collected, which was diluted with Buffer A and centrifuged at 12,000 *g* for 15 min. The pellet was collected and washed three times with Buffer A.

### In-gel protein digestion

Subcellular fractions 2, 4, 6, 8, and 10 were separated on a 12% SDS-PAGE gel and stained overnight with Coomassie G-250 (Invitrogen). Lanes of each fraction were manually cut into three gel bands. In-gel protein digestion was performed as described in the literature (Zhu *et al.* 2018). The tryptic peptides were dried using a SpeedVac and stored at −20 °C for further analysis.

### MS analysis

The nano Liquid chromatography (LC)–MS/MS experiments were performed on a Q Exactive mass spectrometer (Thermo Scientific) coupled to an Easy-nLC 1000 HPLC system (Thermo Scientific). The dried peptides were resuspended in 0.1% formic acid/2% acetonitrile, and loaded onto a 100 μm id × 2 cm fused silica trap column packed in-house with reversed phase silica (Reprosil-Pur C18 AQ, 5 μm, Dr. Maisch GmbH) and then separated on an a 75 μm id × 20 cm C18 column packed with reversed phase silica (Reprosil-Pur C18 AQ, 3 μm, Dr. Maisch GmbH). The loaded peptides were eluted with a 78-min gradient. The solvent A consisted of 0.1% FA in water solution and the solvent B consisted of 0.1% FA in acetonitrile solution. The segmented gradient was 4%–12% B, 5 min; 12%–22% B, 50 min; 22%–32% B, 12 min; 32%–95% B, 1 min; 95% B, 7 min at a flow rate of 280 nl/min.

The mass spectrometer was operated in the data-dependent acquisition mode, and full-scan MS data were acquired in the Orbitrap with a resolution of 70,000 (*m*/*z* 200) across the mass range of 300–1600 *m*/*z*. The target value was 3.00 × 10^6^ with a maximum injection time of 60 ms. After the survey scans, the top 20 most intense precursor ions were selected for MS/MS fragmentation with isolation width of 2 *m*/*z* in the HCD collision cell with optimized normalized collision energy of 32%. Subsequently, MS/MS spectra were acquired in the Orbitrap with a resolution of 17,500 (*m*/*z* 200) and a low mass cut-off setting of 100 *m*/*z*. The target value was set as 5.00 × 10^4^ with a maximum injection time of 80 ms. The dynamic exclusion time was 50 s. For nanoelectrospray ion source setting, the spray voltage was 2.0 kV; no sheath gas flow; the heated capillary temperature was 320 °C.

The raw MS data were processed with Maxquant (v1.5.0.30). The UniProt Rattus norvegicus proteome supplemented with all the frequently observed contaminants in MS served as the database. To search precursor and fragment ions, an initial maximal mass deviation of 10 and 20 ppm, respectively, was required. Tryptic full enzyme specificity with no proline restriction and only peptides with a minimum length of seven amino acids were selected. A maximum of two missed cleavages were allowed. Carbamidomethylation (Cys) was set as the fixed modification. Oxidation (Met) and N-acetylation were considered variable modifications. For identification of protein and peptide, we required a maximum FDR of 1%.

### Western blot

The pellets of enriched granules were prepared in RIPA buffer (50 mmol/L Tris pH 7.4, 150 mmol/L NaCl, 0.1% SDS, 0.5% sodium deoxycholate, 1% Triton X-100, protease cocktail, 1 mmol/L PMSF, 10 mmol/L sodium azide, 10 mmol/L sodium ascorbate, and 5 mmol/L Trolox) for 20 min on the ice. After centrifugation to remove insoluble materials, the protein concentration was determined with the bicinchoninic acid assay (Thermo Scientific, 23227). The sample was diluted with 5× SDS buffer for 10 min at 95 °C, separated by SDS-PAGE and transferred to a nitrocellulose membrane at 400 mA for 60 min in a 10% methanol transfer buffer. The membrane was first blocked for 1 h with 5% non-fat dry milk in TBST and then incubated with the primary antibody overnight at 4 °C. The membrane was washed six times for 5 min each time in TBST, then incubated with the second IgG antibodies conjugated with horseradish peroxidase (HRP) for 1 h at the room temperature, and washed six times for 5 min each time in TBST. Western blotting signals were detected and analyzed using HRP and ChemiScope Touch 6000 (Clinx, Shanghai, China).

### Immunofluorescence

INS-1 cells grown in a glass bottom dish were washed in PBS, then fixed in 4% paraformaldehyde (PFA) for 20 min and permeabilized with 0.2% saponin and 5% albumin bovine in PBS for 1 h at room temperature. Cells were washed two times for 5 min each time and incubated with the primary antibody overnight at 4 °C. After washing six times for 5 min each time, the cells were labeled with secondary antibodies for 1 h (insulin was labeled by TRITC-conjugated goat anti-guinea pig, while TM9SF3 was labeled by Alexa Fluor 488-conjugated goat anti-rabbit). Then, the cells were washed six times for 5 min each time and mounted with DAPI Fluoromount-G. All confocal microscope images were generated using an Olympus FV1200 Laser Scanning Confocal Microscope (Olympus, Tokyo, Japan) with a 100× (NA = 1.40) oil objective.

## Results and discussion

**Fig. 1:**
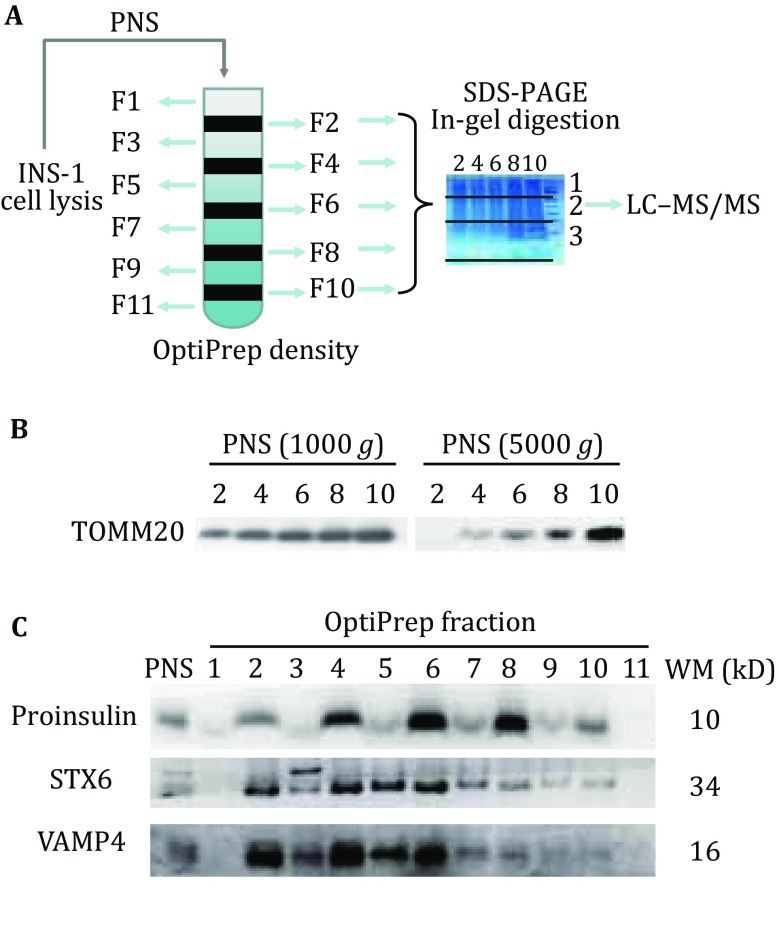
**A** Post-nuclear supernatant separation with OptiPrep density gradient centrifugation, followed by SDS-PAGE, in-gel protein digestion, and peptide analysis by LC–MS/MS. **B** Western blot analysis of TOMM20 in OptiPrep fractions of PNS collected with centrifugation speeds of 1000 and 5000 *g*. **C** Western blot analysis of proinsulin, STX6, and VAMP4 in each OptiPrep fraction. All antibodies were diluted 1:1000

We reported a new strategy for efficient identification of the ISG proteome in INS-1 cells. As shown in Fig. 1A, this strategy includes three steps: (1) enrichment of the ISG fraction by OptiPrep density gradient centrifugation; (2) protein separation by SDS-PAGE followed by in-gel protein digestion and LC–MS/MS analysis; and (3) PCP analysis.

For the enrichment of the ISG fraction, INS-1 cells were lysed and centrifuged at 1000 *g*. The pellets were resuspended and further centrifuged at 5000 *g* to remove most of the mitochondrial fractions or other heavy subcellular organelles. The resulting PNS fraction contained less mitochondria than that prepared by low centrifugation speed, as shown by detection of the mitochondrial marker protein TOMM20 (Fig. 1B). Next, a discontinuous OptiPrep density gradient that had five concentrations (8.8%, 13.2%, 17.6%, 23.4%, and 30%) (Chen *et al.* 2015) was prepared for the separation of PNS. All resulting twelve fractions and raw PNS were assessed by Western blotting experiments to measure the efficiency of OptiPrep separation. As shown in Fig. 1C, proinsulin was mainly detected in fractions 4, 6, and 8. Syntaxin 6 (STX6), which mediates iISG homotypic fusion (Wendler *et al.* 2001), was abundant in fractions 4 and 6. Vesicle-associated membrane protein 4 (VAMP4), which was implicated in TGN vesicle trafficking (Steegmaier *et al.* 1999), was mainly enriched in fractions 2, 4, and 6 and rarely detected in fractions 8 and 10. These results indicated that the ISG proteins were enriched in fractions 4, 6, and 8, and iISG proteins were mainly in fractions 4 and 6.

**Fig. 2:**
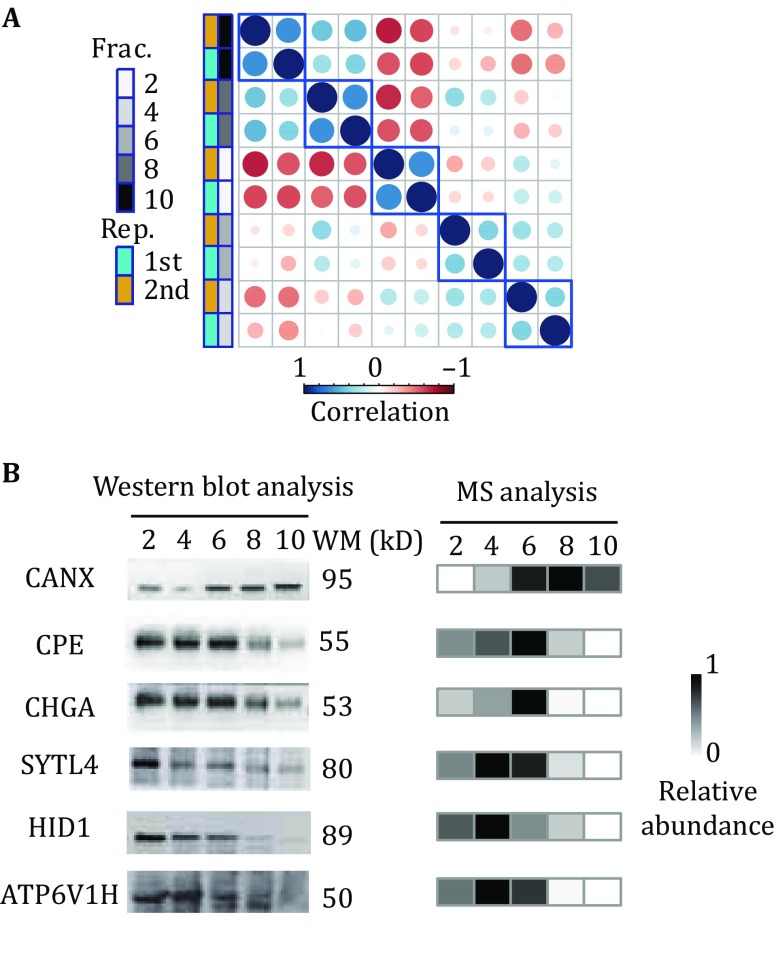
**A** Pearson correlation matrix based on the results of hierarchical clustering of proteins quantified in each OptiPrep fraction by two independent experiments, and blue rectangles indicate the reproducibility of biological replicates. **B** Six proteins (CANX, CPE, CHGA, SYTL4, HID-1, and ATP6V1H) were randomly selected for the comparison of their relative abundances detected by Western blot and MS in OptiPrep fractions 2, 4, 6, 8, and 10. All antibodies were diluted 1:1000

To identify membrane proteins more efficiently, the proteins in each OptiPrep fraction were identified by the use of in-gel digestion-based proteomics workflow (Choksawangkarn *et al.* 2012). All the MS raw data were analyzed by Maxquant software (Cox and Mann 2008). With a false discovery rate of less than 1% at both the peptide and protein levels, 2070 proteins were repeatedly identified by both biological experiments. MS intensity of proteins/protein groups reported by Maxquant was used as the relative quantitative abundance of proteins. To assess the reproducibility of the proteomics experiments, we performed Pearson correlation analysis based on the results of hierarchical clustering of proteins quantified in each OptiPrep fraction. The results showed that proteins in same fraction from two independent experiments were clustered together (Fig. 2A), indicating that our quantitative proteome was highly reproducible. We next performed Western blots to analyze several randomly selected proteins, including CANX, CPE, CHGA, SYTL4, HID-1, and ATP6V1H (Fig. 2B). The results showed that the relative abundance of proteins measured by MS and Western blot was quite comparable, demonstrating the high accuracy of our quantitative proteomic data.

**Fig. 3:**
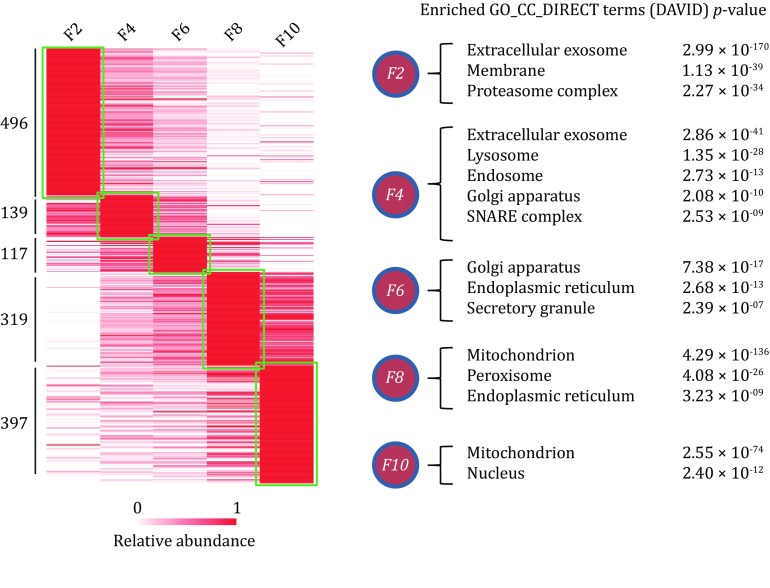
The heatmap of normalized PCP indicating the enrichment of proteins in each OptiPrep fraction. GO cellular component annotation analysis was performed with the DAVID tool. Representative enriched GO terms and their *p* value are listed

Next, we refined the proteome data by excluding the proteins in which the relative abundance correlation between two replicates was less than 0.5. We identified 1468 proteins as the high confidence proteome data for the next PCP analysis. The MS intensities of proteins detected by two replicates were averaged and normalized using the max–min normalization method as quantitative PCP dataset. To analyze the general feature of the proteome in each subcellular fraction, we categorized the proteins based on their PCP distributions into five groups; 496, 139, 117, 319, and 397 proteins with clearly similar abundance patterns were highly enriched in OptiPrep fractions 2, 4, 6, 8, and 10, respectively (Fig. 3). The GO cellular component annotation analysis by DAVID (da Huang *et al*. 2009) revealed that fraction 2 mainly comprised proteins from the extracellular exosome, membrane, and proteasome; fraction 4 mainly comprised proteins from the lysosome, endosome, Golgi apparatus, and SNARE complex; fraction 6 was enriched in proteins from organelles such as the Golgi apparatus, endoplasmic reticulum (ER), and secretory granule; fraction 8 was highly enriched in proteins from the mitochondria, peroxisome and ER, and fraction 10 mainly comprised proteins from the mitochondria and nucleus. Interestingly, we found that ISG-related proteins were highly enriched in fraction 6, including CHGA, PTPRN2, INS1, INS2, PAM, PCSK2, and RAB37, indicating our subcellular fractionation method successfully enriched ISGs in one fraction.

**Fig. 4:**
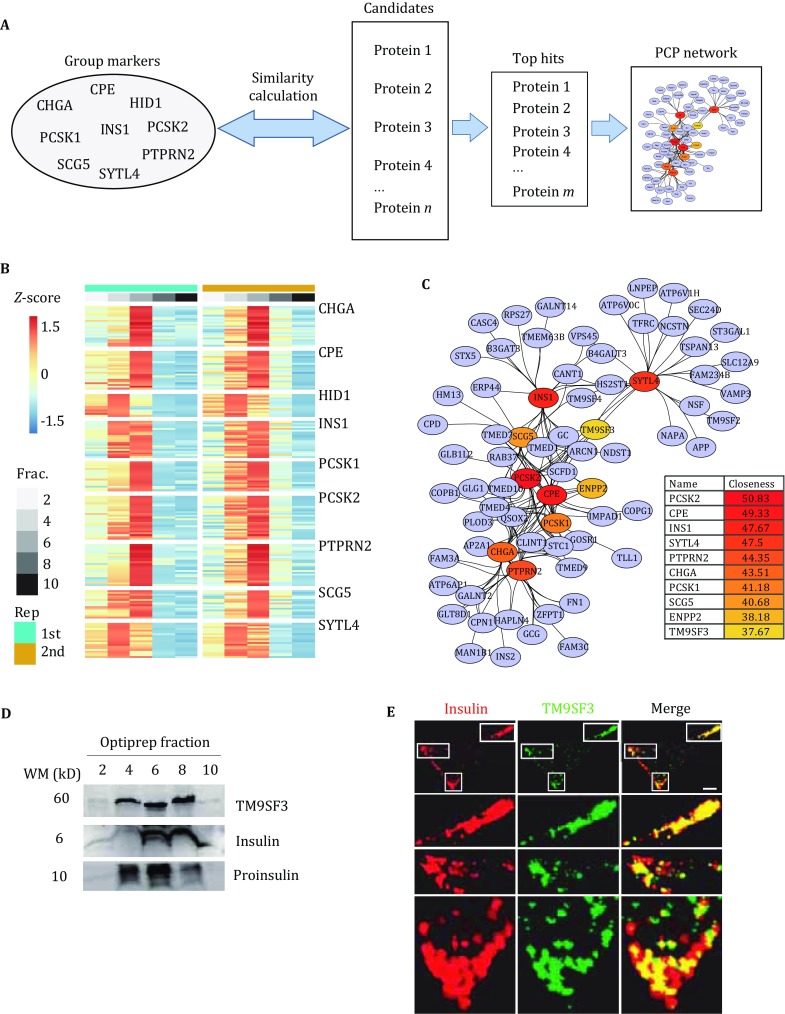
**A** Schematic to illustrate the PCP data analysis. **B** Heatmap of relative abundance of potential ISG proteins. Data were scaled by Z-transformation. **C** PCP network of potential ISG proteins. **D** Western blot analysis of TM9SF3, insulin and proinsulin in OptiPrep fractions. **E** Immunofluorescence micrographs showed the co-localization of TM9SF3 (*green*) and insulin (*red*) in INS-1 cells. Anti-insulin was diluted 1:400, and anti-TM9SF3 was diluted 1:100. Scale bar: 5 μm

For PCP analysis of ISG, the ideal marker proteins of ISGs are proinsulin and insulin, representing iISGs and mISGs, respectively. However, bottom-up-based proteomics techniques cannot distinguish proinsulin, insulin, and C-peptide after tryptic protein digestion because of the common tryptic peptides (Cheng *et al.* 2015). So our MS-measured insulin represented the total amount of protein products encoded by *Ins1* and cannot be used as a reference to dissect the iISGs and mISGs. To address such limitation, we selected another eight known ISG-related proteins, including CHGA (Bandyopadhyay and Mahata 2017), CPE (Cool and Loh 1998; Rindler 1998), HID1 (Du *et al.* 2016), PCSK1 (Furukawa *et al.* 1999), PCSK2 (Guest 2017), PTPRN2 (Caromile *et al.* 2010; Suckale and Solimena 2010), SCG5 (Osamura *et al.* 1988; Tatsumi *et al.* 2003) and SYTL4 (Torii *et al.* 2002; Yi *et al.* 2002). These proteins participated in the distinct steps of ISG maturation process representing the dynamic composition of ISG, so we treated them as a group-marker of ISGs to perform PCP analysis as illustrated in Fig. 4A. ED of purification profiles between the group-marker and each quantified proteins was calculated iteratively. The top 100 lists ranked by ED were recognized as the significant candidates (Supplemental Tables S1, S2). Finally, 81 ISG candidate proteins were repeatedly identified in two biological replicates (Fig. 4B). The PCP network was further built to visualize the relationship between the group-marker and ISG candidates (Fig. 4C). The top ten nodes ranked by the closeness score of the PCP network were highlighted to demonstrate the central nodes. Except for HID-1, all other proteins and the group-marker were top-ranked, indicating the rationality of selecting them as ISG group-markers.

Among 81 ISG proteins identified in our study, several proteins are known ISG-related proteins, including Insulin1/2, CPE, PCSK1, PCSK2, CHGA, ATP6AP1, (Supplemental Tables S1, S2), suggesting the efficiency of our strategy and the reliability of our dataset. During ISG biogenesis, maturation, transportation, and exocytosis, membrane proteins are considered to play essential roles. In our data, more than 60% identified proteins are membrane proteins or membrane-associated proteins. Some of them are potential to function in ISG-related processes, such as ARFRP1, SCFD1, STX12, STX5, VPS45, and ATP6V0C. For example, ATP6V0C is an essential subunit of vacuolar ATPase, a critical enzyme complex that regulates the acidification of the granular lumen which is one of the indispensable steps during ISG maturation (Mangieri *et al.* 2014). SNARE- and SNARE-associated proteins are critical factors for mediating membrane fusion (Advani *et al.* 1998; Laufman *et al.* 2009; Zouhar *et al.* 2009). During ISG maturation, immature ISGs need undergo a homotypic fusion process, in which several SNARE proteins, such as STX12, VTI1A, STX6, and VAMP4, were reported to be involved (Brandhorst *et al.* 2006; Zwilling *et al.* 2007). We speculated STX12 may also play a role in regulating ISG maturation. Taken together, our results suggested that, compared with conventional subcellular fraction method, the PCP-based strategy is more effective for the identification of ISG proteins.

Intriguingly, we noticed that a membrane protein, TM9SF3, was included in the top 10 lists of central nodes (Fig. 4C). For verification, we first determined the protein abundance of TM9SF3 in each OptiPrep fraction. Compared with the distribution of proinsulin and insulin, TM9SF3 distribution was well correlated with insulin content (Fig. 4D). Next, we investigated the cellular distribution of TM9SF3 in INS-1 cells (Fig. 4E). Immunofluorescence results showed that TM9SF3 had good co-localization with insulin, especially in the cell periphery. These results indicated that TM9SF3 may be a new ISG maker. More extensive studies are needed in the future to examine whether TM9SF3 has functions in ISG maturation.

The PCP-based strategy used in the present work can be applicable for ISG functional research by defining the dynamic ISG proteome under different physiological conditions. Furthermore, it can be easily adapted for the proteomic analysis of other highly dynamic organelles, such as endosomes. Our study demonstrated that label-free proteomics was also compatible with PCP analysis, just as other labeling-based methods, such as isobaric labeling and SILAC. Therefore, one can adopt the same strategy to investigate the dynamic proteomes in organelles under multiple stimuli. Since there is no need for labeling protein/peptide, our PCP strategy can be used to identify the organelle proteome from a wide range of sources, including cells and tissues.

## Conclusion

In this study, combined with subcellular fractionation by OptiPrep density gradient centrifugation, we employed a quantitative proteomics approach based on PCP analysis to efficiently measure the ISG proteome. This combination of optimized fractionation and PCP methodology enabled us to extract 81 potential ISG proteins from thousands of co-isolated proteins with high confidence. Several previously known ISG proteins were included in our lists, validating our approach. Further data mining by PCP network analysis enabled us to find and validate a new potential ISG protein, TM9SF3. This strategy can also be easily extended to identify the proteomes of other highly dynamic organelles, such as endosomes, and define the dynamic organelle proteome under different physiological conditions.

## Electronic supplementary material

Below is the link to the electronic supplementary material.

Supplementary material 1 (PDF 74 kb)
