# Supplementary material for: Proteomic analysis of insulin secretory granules in INS-1 cells by protein correlation profiling
Source: Biophys Rep. 2018 Aug 29;4(6):329–38. doi: 10.1007/s41048-018-0061-3 (PMC6276070; doi:10.1007/s41048-018-0061-3)
Supplement: Supplementary file 1 — Supplementary material 1 (PDF 74 kb) [file 41048_2018_61_MOESM1_ESM.pdf]

Supplementary materials

Table S1 The list of ISG potential candidates that were identified using protein correlation profiling by calculating the Euclidean distance between them and ISGs markers (Chga, Cpe, Hid1, Ins1, Pcsk1, Pcsk2, Ptprn2, Scg5, Sytl4)

ISGs Makers	Target	Uniprot	Euclidean distance
Chga	Galnt2	tr|A0A0G2JU25|A0A0G2JU25_RAT;tr|D3ZFD2|D3ZFD2_RAT	0.0569184
Chga	Glg1	sp|Q62638|GSLG1_RAT;tr|G3V8G5|G3V8G5_RAT	0.0698319
Chga	Zfpl1	tr|M0RCA3|M0RCA3_RAT	0.088497
Chga	Ptprn2	sp|Q63475|PTPR2_RAT	0.105461
Chga	Qsox2	tr|D3ZP13|D3ZP13_RAT	0.117665
Chga	Fn1	sp|P04937|FINC_RAT;tr|A0A096P6L8|A0A096P6L8_RAT; tr|F1LST1|F1LST1_RAT	0.134109
Chga	Hapln4	tr|D3Z9H2|D3Z9H2_RAT	0.141703
Chga	Glt8d1	tr|Q7TP03|Q7TP03_RAT;sp|Q6AYF6|GL8D1_RAT	0.150501
Chga	Gosr1	sp|Q62931|GOSR1_RAT	0.156834
Chga	Gcg	tr|G3V6P5|G3V6P5_RAT;sp|P06883|GLUC_RAT	0.190073
Chga	Atp6ap1	sp|O54715|VAS1_RAT;tr|Q6IRF8|Q6IRF8_RAT	0.219245
Chga	Cpn1	sp|Q9EQV8|CBPN_RAT	0.225164
Chga	Pcsk2	sp|P28841|NEC2_RAT	0.235778
Chga	Stc1	sp|P97574|STC1_RAT	0.255162
Chga	Tmed4	tr|G3V6N2|G3V6N2_RAT;tr|A0A0G2K8M5|A0A0G2K8M5_ RAT	0.260677
Chga	Scfd1	sp|Q62991|SCFD1_RAT	0.272812
Chga	Plod3	sp|Q5U367|PLOD3_RAT	0.277956
Chga	Tmed9	sp|Q5I0E7|TMED9_RAT	0.281133
Chga	Tmed10	sp|Q63584|TMEDA_RAT	0.28352
Chga	Cpe	tr|D4A8X4|D4A8X4_RAT;sp|P15087|CBPE_RAT	0.291278
Chga	Ins2	sp|P01323|INS2_RAT	0.29236
Chga	Fam3a	tr|B5DFJ2|B5DFJ2_RAT	0.306231
Cpe	Stc1	sp|P97574|STC1_RAT	0.0569577
Cpe	Enpp2	sp|Q64610|ENPP2_RAT;tr|F1LSF4|F1LSF4_RAT;tr|D3ZES5| D3ZES5_RAT	0.0629894
Cpe	Arcn1	sp|Q66H80|COPD_RAT	0.0717812
Cpe	Pcsk2	sp|P28841|NEC2_RAT	0.0744404
Cpe	Impad1	tr|Q7TPJ5|Q7TPJ5_RAT;sp|D4AD37|IMPA3_RAT	0.123147
Cpe	Copb1	sp|P23514|COPB_RAT	0.12432
Cpe	Gc	tr|Q68FY4|Q68FY4_RAT;sp|P04276|VTDB_RAT	0.128805
Cpe	Pcsk1	tr|A0A0G2JX54|A0A0G2JX54_RAT;sp|P28840|NEC1_RAT	0.142568
Cpe	Ins1	sp|P01322|INS1_RAT	0.152098
Cpe	Sytl4	sp|Q8VHQ7|SYTL4_RAT	0.164674
Cpe	Clint1	tr|A0A0G2JW94|A0A0G2JW94_RAT;tr|Q6DGF2|Q6DGF2_ RAT	0.166501
Cpe	Gosr1	sp|Q62931|GOSR1_RAT	0.187481

Cpe	Copg1	sp|Q4AEF8|COPG1_RAT;tr|A0A0G2K1F3|A0A0G2K1F3_RAT	0.19069
Cpe	Tm9sf3	tr|D3ZUD8|D3ZUD8_RAT	0.190761
Cpe	Qsox2	tr|D3ZP13|D3ZP13_RAT	0.193194
Cpe	Ap2a1	tr|D3ZUY8|D3ZUY8_RAT	0.206744
Cpe	Rab37	tr|D4A0G7|D4A0G7_RAT	0.208311
Cpe	Ptprn2	sp|Q63475|PTPR2_RAT	0.213242
Cpe	Plod3	sp|Q5U367|PLOD3_RAT	0.220945
Cpe	Tmed4	tr|G3V6N2|G3V6N2_RAT;tr|A0A0G2K8M5|A0A0G2K8M5_RAT	0.221403
Cpe	Scg5	tr|G3V714|G3V714_RAT;sp|P27682|7B2_RAT	0.221507
Hid1	Tmem30a	tr|A0A0G2JXG3|A0A0G2JXG3_RAT;sp|Q6AY41|CC50A_RAT	0.132401
Hid1	Ncstn	tr|B3DM90|B3DM90_RAT;sp|Q8CGU6|NICA_RAT	0.140366
Hid1	Sec24d	tr|G3V9S9|G3V9S9_RAT	0.143599
Hid1	Napa	sp|P54921|SNAA_RAT	0.149335
Hid1	Stx12	tr|A0A0G2JVB6|A0A0G2JVB6_RAT;sp|G3V7P1|STX12_RAT;tr|A0A0G2K4U8|A0A0G2K4U8_RAT	0.167116
Hid1	Lamtor3	sp|Q5U204|LTOR3_RAT	0.176782
Hid1	Cnp	sp|P13233|CN37_RAT	0.181571
Hid1	Arfrp1	sp|Q63055|ARFRP_RAT	0.182491
Hid1	Stambp	sp|Q8R424|STABP_RAT	0.188186
Hid1	Lamp1	sp|P14562|LAMP1_RAT	0.193812
Hid1	Rab11a	sp|P62494|RB11A_RAT;tr|A0A0G2JZR4|A0A0G2JZR4_RAT;sp|O35509|RB11B_RAT	0.2052
Hid1	Lnpep	sp|P97629|LCAP_RAT;tr|A0A0H2UHK5|A0A0H2UHK5_RAT;tr|A0A0G2JUW8|A0A0G2JUW8_RAT;tr|A0A0G2JYN3|A0A0G2JYN3_RAT	0.206915
Ins1	Tmed10	sp|Q63584|TMEDA_RAT	0.128213
Ins1	Rab37	tr|D4A0G7|D4A0G7_RAT	0.134912
Ins1	Tmed7	sp|D3ZTX0|TMED7_RAT	0.15524
Ins1	Scfd1	sp|Q62991|SCFD1_RAT	0.162747
Ins1	Arcn1	sp|Q66H80|COPD_RAT	0.162767
Ins1	Casc4	tr|D3ZW58|D3ZW58_RAT	0.187641
Ins1	Rps27	sp|Q71TY3|RS27_RAT;tr|M0RA26|M0RA26_RAT	0.189074
Ins1	Tmem63b	tr|D4A105|D4A105_RAT	0.193845
Ins1	Tmed1	sp|Q5BK85|TMED1_RAT	0.195676
Ins1	Enpp2	sp|Q64610|ENPP2_RAT;tr|F1LSF4|F1LSF4_RAT;tr|D3ZES5|D3ZES5_RAT	0.201641
Ins1	Vps45	sp|O08700|VPS45_RAT	0.210855
Ins1	Sytl4	sp|Q8VHQ7|SYTL4_RAT	0.216534
Ins1	Hs2st1	tr|G3V7N0|G3V7N0_RAT	0.218947
Ins1	Tm9sf3	tr|D3ZUD8|D3ZUD8_RAT	0.219586
Ins1	Galnt14	tr|Q6AYA4|Q6AYA4_RAT	0.223466
Ins1	B3gat3	tr|B2GV35|B2GV35_RAT	0.227941
Ins1	Stx5	sp|Q08851|STX5_RAT	0.228368
Ins1	B4galt3	sp|Q6P768|B4GT3_RAT	0.229702
Ins1	Gc	tr|Q68FY4|Q68FY4_RAT;sp|P04276|VTDB_RAT	0.229845

Ins1	Erp44	tr|Q5VLR5|Q5VLR5_RAT	0.237435
Pcsk1	Clint1	tr|A0A0G2JW94|A0A0G2JW94_RAT;tr|Q6DGF2|Q6DGF2_RAT	0.105733
Pcsk1	Gc	tr|Q68FY4|Q68FY4_RAT;sp|P04276|VTDB_RAT	0.116375
Pcsk1	Impad1	tr|Q7TPJ5|Q7TPJ5_RAT;sp|D4AD37|IMPA3_RAT	0.129362
Pcsk1	Enpp2	sp|Q64610|ENPP2_RAT;tr|F1LSF4|F1LSF4_RAT;tr|D3ZES5|D3ZES5_RAT	0.138934
Pcsk1	Rab37	tr|D4A0G7|D4A0G7_RAT	0.14044
Pcsk1	Cpe	tr|D4A8X4|D4A8X4_RAT;sp|P15087|CBPE_RAT	0.142568
Pcsk1	Stc1	sp|P97574|STC1_RAT	0.16125
Pcsk1	Tmed10	sp|Q63584|TMEDA_RAT	0.170855
Pcsk1	Pcsk2	sp|P28841|NEC2_RAT	0.171285
Pcsk1	Tmed4	tr|G3V6N2|G3V6N2_RAT;tr|A0A0G2K8M5|A0A0G2K8M5_RAT	0.17687
Pcsk1	Tmed1	sp|Q5BK85|TMED1_RAT	0.178405
Pcsk1	Gosr1	sp|Q62931|GOSR1_RAT	0.18601
Pcsk1	Tmed7	sp|D3ZTX0|TMED7_RAT	0.190257
Pcsk1	Arcn1	sp|Q66H80|COPD_RAT	0.191491
Pcsk1	Qsox2	tr|D3ZP13|D3ZP13_RAT	0.207429
Pcsk1	Tll1	tr|D3Z8U5|D3Z8U5_RAT	0.211053
Pcsk2	Cpe	tr|D4A8X4|D4A8X4_RAT;sp|P15087|CBPE_RAT	0.0744404
Pcsk2	Stc1	sp|P97574|STC1_RAT	0.0855415
Pcsk2	Arcn1	sp|Q66H80|COPD_RAT	0.104144
Pcsk2	Ins1	sp|P01322|INS1_RAT	0.120378
Pcsk2	Enpp2	sp|Q64610|ENPP2_RAT;tr|F1LSF4|F1LSF4_RAT;tr|D3ZES5|D3ZES5_RAT	0.13147
Pcsk2	Gosr1	sp|Q62931|GOSR1_RAT	0.135317
Pcsk2	Qsox2	tr|D3ZP13|D3ZP13_RAT	0.146571
Pcsk2	Ptprn2	sp|Q63475|PTPR2_RAT	0.15023
Pcsk2	Plod3	sp|Q5U367|PLOD3_RAT	0.159383
Pcsk2	Scg5	tr|G3V714|G3V714_RAT;sp|P27682|7B2_RAT	0.16188
Pcsk2	Pcsk1	tr|A0A0G2JX54|A0A0G2JX54_RAT;sp|P28840|NEC1_RAT	0.171285
Pcsk2	Impad1	tr|Q7TPJ5|Q7TPJ5_RAT;sp|D4AD37|IMPA3_RAT	0.177781
Pcsk2	Glb1l2	tr|D3ZHQ5|D3ZHQ5_RAT	0.178107
Pcsk2	Tmed4	tr|G3V6N2|G3V6N2_RAT;tr|A0A0G2K8M5|A0A0G2K8M5_RAT	0.183778
Pcsk2	Gc	tr|Q68FY4|Q68FY4_RAT;sp|P04276|VTDB_RAT	0.195167
Pcsk2	Scfd1	sp|Q62991|SCFD1_RAT	0.198284
Pcsk2	Tmed10	sp|Q63584|TMEDA_RAT	0.205116
Pcsk2	Sytl4	sp|Q8VHQ7|SYTL4_RAT	0.208412
Pcsk2	Glg1	sp|Q62638|GSLG1_RAT;tr|G3V8G5|G3V8G5_RAT	0.212342
Pcsk2	Rab37	tr|D4A0G7|D4A0G7_RAT	0.212579
Pcsk2	Clint1	tr|A0A0G2JW94|A0A0G2JW94_RAT;tr|Q6DGF2|Q6DGF2_RAT	0.215882
Pcsk2	Tm9sf3	tr|D3ZUD8|D3ZUD8_RAT	0.217874
Pcsk2	Tmed7	sp|D3ZTX0|TMED7_RAT	0.223314
Pcsk2	Tmed9	sp|Q5I0E7|TMED9_RAT	0.224433
Ptprn2	Qsox2	tr|D3ZP13|D3ZP13_RAT	0.0587142

Ptprn2	Gosr1	sp|Q62931|GOSR1_RAT	0.0628878
Ptprn2	Zfpl1	tr|M0RCA3|M0RCA3_RAT	0.0634938
Ptprn2	Man1b1	sp|B2GUY0|MA1B1_RAT	0.0850438
Ptprn2	Galnt2	tr|A0A0G2JU25|A0A0G2JU25_RAT;tr|D3ZFD2|D3ZFD2_RAT	0.105425
Ptprn2	Chga	sp|P10354|CMGA_RAT;tr|Q5PPG5|Q5PPG5_RAT	0.105461
Ptprn2	Gcg	tr|G3V6P5|G3V6P5_RAT;sp|P06883|GLUC_RAT	0.119256
Ptprn2	Glt8d1	tr|Q7TP03|Q7TP03_RAT;sp|Q6AYF6|GL8D1_RAT	0.121161
Ptprn2	Hapln4	tr|D3Z9H2|D3Z9H2_RAT	0.127466
Ptprn2	Pcsk2	sp|P28841|NEC2_RAT	0.15023
Ptprn2	Cpn1	sp|Q9EQV8|CBPN_RAT	0.161079
Ptprn2	Tmed4	tr|G3V6N2|G3V6N2_RAT;tr|A0A0G2K8M5|A0A0G2K8M5_RAT	0.167965
Ptprn2	Fn1	sp|P04937|FINC_RAT;tr|A0A096P6L8|A0A096P6L8_RAT;tr| F1LST1|F1LST1_RAT	0.170587
Ptprn2	Stc1	sp|P97574|STC1_RAT	0.193266
Ptprn2	Tmed10	sp|Q63584|TMEDA_RAT	0.195489
Ptprn2	Plod3	sp|Q5U367|PLOD3_RAT	0.199308
Ptprn2	Atp6ap1	sp|O54715|VAS1_RAT;tr|Q6IRF8|Q6IRF8_RAT	0.201547
Ptprn2	Tmed9	sp|Q5I0E7|TMED9_RAT	0.208727
Ptprn2	Cpe	tr|D4A8X4|D4A8X4_RAT;sp|P15087|CBPE_RAT	0.213242
Ptprn2	Pcsk1	tr|A0A0G2JX54|A0A0G2JX54_RAT;sp|P28840|NEC1_RAT	0.242089
Ptprn2	Fam3a	tr|B5DFJ2|B5DFJ2_RAT	0.243979
Ptprn2	Enpp2	sp|Q64610|ENPP2_RAT;tr|F1LSF4|F1LSF4_RAT;tr|D3ZES5|D3ZES5_RAT	0.251439
Ptprn2	Fam3c	tr|A0A0G2JUB0|A0A0G2JUB0_RAT;sp|Q810F4|FAM3C_RAT	0.260851
Scg5	Cpd	tr|F1LPC6|F1LPC6_RAT;sp|Q9JHW1|CBPD_RAT;tr|A0A0G2JY30|A0A0G2JY30_RAT	0.101218
Scg5	Glg1	sp|Q62638|GSLG1_RAT;tr|G3V8G5|G3V8G5_RAT	0.11014
Scg5	Pcsk2	sp|P28841|NEC2_RAT	0.16188
Scg5	Qsox2	tr|D3ZP13|D3ZP13_RAT	0.179803
Scg5	Ndst1	sp|Q02353|NDST1_RAT	0.197975
Scg5	Cpe	tr|D4A8X4|D4A8X4_RAT;sp|P15087|CBPE_RAT	0.221507
Scg5	Scfd1	sp|Q62991|SCFD1_RAT	0.225001
Scg5	Arcn1	sp|Q66H80|COPD_RAT	0.23407
Scg5	Ins1	sp|P01322|INS1_RAT	0.249787
Scg5	Enpp2	sp|Q64610|ENPP2_RAT;tr|F1LSF4|F1LSF4_RAT;tr|D3ZES5|D3ZES5_RAT	0.26516
Scg5	Tm9sf4	sp|Q4KLL4|TM9S4_RAT;tr|A0A0G2KA25|A0A0G2KA25_RAT	0.268902
Scg5	Hm13	tr|D3ZP98|D3ZP98_RAT;tr|A0A0G2K1Z9|A0A0G2K1Z9_RAT	0.26983
Scg5	Tmed4	tr|G3V6N2|G3V6N2_RAT;tr|A0A0G2K8M5|A0A0G2K8M5_RAT	0.280409
Scg5	Cant1	sp|Q8K4Y7|CANT1_RAT	0.28343
Scg5	Erp44	tr|Q5VLR5|Q5VLR5_RAT	0.295744
Sytl4	Fam234b	tr|D3ZWJ9|D3ZWJ9_RAT	0.0384438
Sytl4	Vps45	sp|O08700|VPS45_RAT	0.0424586
Sytl4	App	tr|Q6P6Q5|Q6P6Q5_RAT;sp|P08592|A4_RAT	0.0812737

Sytl4	Tspan13	sp|Q5FVL6|TSN13_RAT	0.103997
Sytl4	St3gal1	tr|E9PSJ1|E9PSJ1_RAT	0.107136
Sytl4	Tm9sf3	tr|D3ZUD8|D3ZUD8_RAT	0.108053
Sytl4	Sec24d	tr|G3V9S9|G3V9S9_RAT	0.111641
Sytl4	Lnpep	sp|P97629|LCAP_RAT;tr|A0A0H2UHK5|A0A0H2UHK5_RAT;tr|A0A0G2JUW8|A0A0G2JUW8_RAT;tr|A0A0G2JYN3|A0A0G2JYN3_RAT	0.115862
Sytl4	Atp6v1h	tr|A0A0G2K9J2|A0A0G2K9J2_RAT;tr|E9PTI1|E9PTI1_RAT	0.129631
Sytl4	Vamp3	sp|P63025|VAMP3_RAT	0.154318
Sytl4	Tfrc	tr|G3V679|G3V679_RAT;sp|Q99376|TFR1_RAT	0.155107
Sytl4	Ncstn	tr|B3DM90|B3DM90_RAT;sp|Q8CGU6|NICA_RAT	0.155809
Sytl4	Cant1	sp|Q8K4Y7|CANT1_RAT	0.164371
Sytl4	Tm9sf4	sp|Q4KLL4|TM9S4_RAT;tr|A0A0G2KA25|A0A0G2KA25_RAT	0.164508
Sytl4	Napa	sp|P54921|SNAA_RAT	0.174195
Sytl4	Atp6v0c	sp|P63081|VATL_RAT	0.174539
Sytl4	Tm9sf2	sp|Q66HG5|TM9S2_RAT	0.179875
Sytl4	Nsf	tr|F1LQ81|F1LQ81_RAT;sp|Q9QUL6|NSF_RAT;tr|A0A0G2K6U1|A0A0G2K6U1_RAT	0.19556
Sytl4	Slc12a9	tr|M0RAK3|M0RAK3_RAT;tr|M0R3V5|M0R3V5_RAT;tr|A0A096MJB7|A0A096MJB7_RAT;sp|Q66HR0|S12A9_RAT	0.197675

Table S2 The list of 81 ISG candidates including their protein names, annotations by the previous studies, and sub-localization based on Uniprot database

Target	Names	Schvartz *et al*. 2012	Brunner et *al*. 2007	Hickey *et al*. 2009	Localization
Chga	Chromogranin-A	X	X	X	Intravesicular protein
Cpe	Carboxypeptidase E	X	X	X	Intravesicular protein
Ins1	Insulin-1	X	X	X	Intravesicular protein
Ins2	Insulin-2	X	X	X	Intravesicular protein
Enpp2	Ectonucleotide pyrophosphatase/ phosphodiesterase family member 2	X	X		Intravesicular protein
Fn1	Fibronectin	X	X		Intravesicular protein
Gc	Group specific component	X	X		Intravesicular protein
Stc1	Stanniocalcin-1	X	X		Intravesicular protein
Plod3	Procollagen-lysine,2-oxoglutarate 5-dioxygenase 3	X	X		Membrane protein
Ptprn2	Receptor-type tyrosine-protein phosphatase N2	X	X		Membrane protein
Vamp3	Vesicle-associated Menbrane protein 3	X	X		Membrane protein
Gcg	Glucagon	X			Intravesicular protein
Pcsk1	Neuroendocrine convertase 1	X			Intravesicular protein
Qsox2	Sulfhydryl oxidase	X			Intravesicular protein
Atp6ap1	V-type proton ATPase subunit S1	X			Membrane protein
Arcn1	Coatomer subunit delta	X			Intravesicular protein
St3gal1	Protein St3gal1	X			Membrane protein
Cnp	2',3'-cyclic-nucleotide 3'-phosphodiesterase	X			Intravesicular protein
Lamp1	Lysosome-associated Menbrane glycoprotein 1	X			Lysosome
Nsf	N-ethylmaleimide sensitive fusion protein, isoform CRA_b	X			Cytoplasm
B3gat3	Galactosylgalactosylxylosyl protein 3-beta-glucuronosyltransferase	X			Membrane protein
Rps27	40S ribosomal protein S27	X			Other
Rab11a	Ras-related protein Rab-11A	X			Membrane protein

Slc12a9	Solute carrier family 12 member 9	X			Membrane protein
Cpd	Carboxypeptidase D	X			Membrane protein
Tfrc	Transferrin receptor protein 1	X			Membrane protein
App	Amyloid beta A4 protein	X			Membrane protein/ Menbrane-assoicated protein
Tmed9	TransMenbrane emp24 domain-containing protein 9	X			Menbrane-assoicated protein
Tmed10	TransMenbrane emp24 domain-containing protein 10			X	Menbrane-assoicated protein
Atp6v1h	Protein Atp6v1h		X		Membrane protein
Cpn1	Carboxypeptidase N catalytic chain		X		Intravesicular protein
Glb1l2	Beta-galactosidase		X		Intravesicular protein
Pcsk2	Neuroendocrine convertase 2		X		Intravesicular protein
Scg5	Neuroendocrine protein 7B2		X		Intravesicular protein
Sytl4	Synaptotagmin-like protein 4		X		Membrane protein
Rab37	Protein Rab37		X		Membrane protein/ Menbrane-assoicated protein
Ap2a1	Adaptor protein complex AP-2, alpha 1 subunit (Predicted)				Membrane protein
Arfrp1	ADP-ribosylation factor-related protein 1				Menbrane-assoicated protein
Atp6v0c	V-type proton ATPase 16 kDa proteolipid subunit				Membrane protein
B4galt3	Beta-1,4-galactosyltransferase 3				Menbrane-assoicated protein
Cant1	Soluble calcium-activated nucleotidase 1				Menbrane-assoicated protein
Casc4	Protein Casc4				Membrane protein
Clint1	Protein Clint1				Menbrane-assoicated protein
Copb1	Coatomer subunit beta				Intravesicular protein
Copg1	Coatomer subunit gamma-1				Intravesicular protein
Erp44	BWK4				ER

Fam234b	Protein Fam234b				Other
Fam3a	Fam3a protein				Menbrane-assoicated protein
Fam3c	Protein FAM3C				Menbrane-assoicated protein
Galnt14	Polypeptide N-acetylgalactosaminyltransferase				Menbrane-assoicated protein
Galnt2	Polypeptide N-acetylgalactosaminyltransferase				Menbrane-assoicated protein
Glg1	Golgi apparatus protein 1				Menbrane-assoicated protein
Glt8d1	Da2-24				Membrane protein
Gosr1	Golgi SNAP receptor complex member 1				Membrane protein
Hapln4	Hyaluronan and proteoglycan link protein 4 (Predicted)				Menbrane-assoicated protein
Hm13	Histocompatibility 13 (Predicted), isoform CRA_a				Membrane protein
Hs2st1	Heparan sulfate 2-O-sulfotransferase 1, isoform CRA_a				Membrane protein
Impad1	Ac2-190				Membrane protein
Lamtor3	Ragulator complex protein LAMTOR3				Menbrane-assoicated protein
Lnpep	Leucyl-cystinyl aminopeptidase				Membrane protein
Man1b1	Endoplasmic reticulum mannosyl-oligosaccharide 1,2-alpha-mannosidase				ER
Napa	Alpha-soluble NSF attachment protein				Membrane protein
Ncstn	Ncstn protein				Membrane protein
Ndst1	Bifunctional heparan sulfate N-deacetylase/ N-sulfotransferase 1				Menbrane-assoicated protein
Scfd1	Sec1 family domain-containing protein 1				Menbrane-assoicated protein
Sec24d	Protein Sec24d				Membrane protein
Stambp	STAM-binding protein				Membrane protein
Stx12	Syntaxin-12				Menbrane-assoicated protein

Stx5	Syntaxin-5				Menbrane-assoicated protein
Tll1	Metalloendopeptidase				Other
Tm9sf2	TransMenbrane 9 superfamily member 2				Membrane protein
Tm9sf3	TransMenbrane 9 superfamily member				Membrane protein
Tm9sf4	TransMenbrane 9 superfamily member 4				Membrane protein
Tmed1	TransMenbrane emp24 domain-containing protein 1				Menbrane-assoicated protein
Tmed4	Protein Tmed4				Menbrane-assoicated protein
Tmed7	TransMenbrane emp24 domain-containing protein 7				Menbrane-assoicated protein
Tmem30a	Cell cycle control protein 50A				Membrane protein
Tmem63b	Protein Tmem63b				Membrane protein
Tspan13	Tetraspanin-13				Membrane protein
Vps45	Vacuolar protein sorting-associated protein 45				Membrane protein/ Menbrane-assoicated protein
Zfpl1	Protein Zfpl1				Membrane protein/ Menbrane-assoicated protein

“X” indicates that the candidate is reported in the literatures.
